# Neurogenic Bladder Physiology, Pathogenesis, and Management after Spinal Cord Injury

**DOI:** 10.3390/jpm12060968

**Published:** 2022-06-14

**Authors:** Nathalie Elisabeth Perez, Neha Pradyumna Godbole, Katherine Amin, Raveen Syan, David R. Gater

**Affiliations:** 1 University of Miami Miller School of Medicine, Miami, FL 33136, USA; nathalieperez@med.miami.edu (N.E.P.); ngodbole@med.miami.edu (N.P.G.); 2 Department of Urology, University of Miami Miller School of Medicine, Miami, FL 33136, USA; katherine.amin@med.miami.edu; 3 Department of Physical Medicine and Rehabilitation, University of Miami Miller School of Medicine, Miami, FL 33136, USA; dgater@miami.edu; 4 The Miami Project to Cure Paralysis, University of Miami Miller School of Medicine, Miami, FL 33136, USA; 5 Christine E. Lynn Rehabilitation Center for the Miami Project to Cure Paralysis, Miami, FL 33136, USA

**Keywords:** spinal cord injury, tetraplegia, paraplegia, neurogenic bladder, autonomic dysreflexia, detrusor sphincter dyssynergia, urinary incontinence

## Abstract

Urinary incontinence is common after spinal cord injury (SCI) due to loss of supraspinal coordination and unabated reflexes in both autonomic and somatic nervous systems; if unchecked, these disturbances can become life-threatening. This manuscript will review normal anatomy and physiology of the urinary system and discuss pathophysiology secondary to SCI. This includes a discussion of autonomic dysreflexia, as well as its diagnosis and management. The kidneys and the ureters, representing the upper urinary tract system, can be at risk related to dyssynergy between the urethral sphincters and high pressures that lead to potential vesicoureteral reflux, urinary tract infections, and calculi associated with neurogenic lower urinary tract dysfunction (NLUTD). Recent guidelines for diagnosis, evaluation, treatment and follow up of the neurogenic bladder will be reviewed and options provided for risk stratification and management. Mechanical, pharmacological, neurolysis and surgical management will be discussed.

## 1. Introduction

Currently, there are 282,000 patients in the U.S. living with spinal cord injury, with 54 new cases per million annually [1,2]. Neurogenic bladder is a common complication and presents differently based on the location of the lesion. Diagnostics include a combination of non-invasive methods, with emphasis on the history and physical as well as invasive options with urodynamic testing and cystoscopy. A variety of non-surgical and surgical management options are utilized for neurogenic bladder with the main goal of preventing renal impairment. 

A review of the literature was performed to identify the most updated physiological and diagnostic information regarding neurogenic bladder after SCI. When available, we utilized the most recent guidelines on the management of neurogenic bladder to describe management and surveillance strategies. We included studies examining interventions and complication outcomes, as well as systematic reviews to provide the most up-to-date information and guidance possible. Additionally, we included studies that focused on quality-of-life metrics to present the patient perspective. 

## 2. Basic Anatomy in the Normal Functioning Urinary Tract

The urinary system is generally divided into an upper and lower tract. In a normal anatomic system, the upper tract is composed of two kidneys and the ureters, while the lower tract is composed of a small portion of the distal ureters, bladder, and urethra. The upper urinary tract has a retroperitoneal location cephalad to the level of T12-L3. The role of the kidneys is to filter water, electrolytes, and waste from the blood, creating urine in the process. This urine is then drained via the ureters to the bladder for storage. The kidneys are innervated by the renal plexus, which is composed of efferent and afferent fibers from the celiac and mesenteric plexus and splanchnic nerves. The ureters are innervated by a ureteric plexus from T12–L2 and receive parasympathetic innervation from S2–S4. 

**Figure 1:**
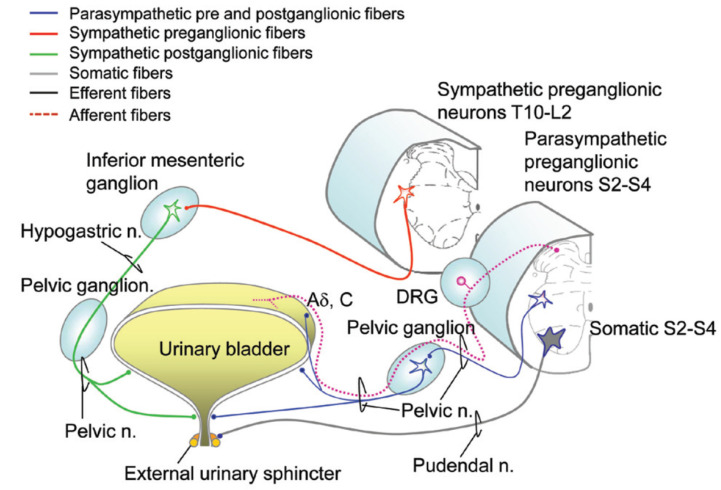
Innervation of the lower urinary tract. DRG: dorsal root ganglion; Aδ and C stretch fibers: mechanosensitive afferents. Adapted with permission from Ref. [4]. 2021, Wecht et al.

The bladder is a viscous organ with a strong smooth muscle wall, called the detrusor muscle, which distends when filled with urine. The bladder has an intraabdominal location until approximately age 6, when the bladder then lies in the pelvis. An internal urethral sphincter controls the involuntary flow of urine from the bladder to the urethra. This is well identified in males; however, it is not readily evident in females. The external urethral sphincter controls the voluntary urinary flow. Sympathetic nervous system (SNS) innervation arises from T10–L2 with the hypogastric nerve, which mediates bladder filling through the activation of α_1_ adrenergic receptors at the bladder neck and β_3_ adrenergic receptors on the detrusor dome, causing relaxation of the body of the detrusor (Figure 1, Table 1) [3,4]. Parasympathetic nervous system (PNS) activity to the bladder is mediated through the pelvic splanchnic nerves that arise from S2–S4, causing bladder emptying primarily due to its effect on detrusor M_3_ cholinergic receptors (Figure 1, Table 1) [3,4]. The pudendal nerve also arises from S2–S4 and innervates the external urethral sphincter and pelvic floor muscles under voluntary, somatic control (Figure 1, Table 1) [3,4]. 

## 3. Normal Physiology in the Functioning Urinary Tract

Coordinated communication between the pontine micturition center, the sacral spinal cord, and the lower urinary tract itself is essential for the control of voiding when deemed appropriate. The pontine micturition center (PMC) is located in the pons and maintains supraspinal control over the detrusor and internal urethral sphincter muscles, allowing for detrusor muscle contraction and internal urethral sphincter relaxation [5]. The PMC, when excited, triggers a voiding reflex. However, the frontal cortex of the brain maintains tonic inhibition of the PMC to allow for bladder storage until it is deemed appropriate to void.

### 3.1. Storage Phase

As the bladder fills, Aδ stretch and C pain receptors on the detrusor muscle send slow signals to the sacral spinal cord at the S2–S4 level via the afferent pelvic nerve. These signals are relayed up to the PMC and higher cortical structures in the brain to notify the brain that the bladder is filling or that there is a desire to urinate [5]. If determined to be an inappropriate time to void, the cortex will maintain inhibition of the PMC, which in turn inhibits PNS activity at the detrusor muscle and internal urethral sphincter via the pelvic splanchnic nerves. Conversely, the PMC will activate the SNS hypogastric nerve to cause detrusor relaxation and internal urethral sphincter contraction to allow for bladder filling. The external urethral sphincter and pelvic floor musculature are innervated by the somatic pudendal nerve and together, with the internal urethral sphincter, help maintain continence during the storage phase by maintaining a higher pressure in the urethra than in the bladder; this is called the guarding reflex [6].

### 3.2. Voiding Phase

With increasing amounts of volume in the bladder, Aδ stretch and C pain receptors in the detrusor muscle activate the afferent pelvic nerve to send faster signals to the sacral spinal cord and brain. The frontal cortex will then release its tonic inhibition on the PMC, if detected to be an appropriate time and place. The PMC will then release its inhibition on the voiding and guarding reflexes, decreasing SNS from hypogastric nerve to relax the bladder neck and internal urethral sphincter, increasing PNS activation along the pelvic splanchnic nerves to activate detrusor muscle contraction, with voluntary somatic withdrawal of the pudendal nerve to the external urethral sphincter [7]. The voiding reflex is an autonomic spinal reflex arc between the detrusor muscle and the sacral spinal cord. Once voiding begins, the reflex arc will ensue, and the detrusor muscle continues to contract to allow for voiding and bladder emptying [7].

#### Spinal Cord Injury

When a spinal cord injury is sustained, the location of the lesion can be described as suprasacral, mixed or sacral/infrasacral [8]. In a suprasacral SCI, the sacral reflex arc and PMC remain intact, but the SCI prevents communication between them, essentially disinhibiting the sacral micturition reflex arc, due to the upper motor neuron (UMN) lesion. The detrusor becomes hyperreflexic, as does the external urethral sphincter, leading to detrusor sphincter dyssynergia (DSD) and very high pressures within the detrusor, putting upper urinary tracts at risk [8,9]. In a mixed SCI, such as conus medullaris syndrome involving both cord and cauda equina, supraspinal disinhibition of the sacral micturition reflex may occur, or may be abolished due to damage to the sacral roots required for the micturition reflex; both conditions can lead to urinary incontinence [8,10]. In a sacral/infrasacral SCI, the lower motor neurons are damaged, and the voiding reflex arc is interrupted while the PMC remains intact, leading to an areflexic detrusor muscle and flaccid external urethral sphincter. Due to the coordinated effects of the PMC, spinal cord, and lower urinary tract, the location of the lesion can help characterize the typical symptoms and diagnostic information observed in these patients. In Table 2, the level of spinal cord injury and its associated type of bladder dysfunction is depicted.

Evidently, the level and completeness of SCI needs to be carefully determined and documented according the most recent International Standards for the Neurological Classification of Spinal Cord Injury (ISNCSCI) [11]. Additionally, the International Standards to determine remaining Autonomic Function after Spinal Cord injury (ISAFSCI) should be completed to determine the risk of autonomic dysfunction [4]. Of paramount importance, individuals with SCI above T6 are at high risk for autonomic dysreflexia (AD), a hypertensive crisis brought on by noxious stimuli below the level of SCI, with reflex sympathetic outflow to the splanchnic vascular bed that is uninhibited by supraspinal influence [4,12]. The greater splanchnic nerve typically arises from T7 and T8 spinal nerve roots; those with T7 SCI and below usually have autonomic control over the splanchnic vascular bed; exceptions occur due to the variable anatomy of the greater splanchnic nerve in some individuals. Of note, the nerve to the adrenal medulla arises from T7 and T8 nerve roots, such that reflex sympathetic activation above this level can cause unchecked epinephrine secretion from the adrenal medulla for those with SCI above this level. Individuals with high thoracic and cervical SCI will usually have baseline neurogenic orthostatic hypotension with systolic blood pressure (SBP) between 90 and 110 mmHg due to sympathetic blunting, as the sympathetic nervous system arises from the thoracolumbar regions of the spinal cord [3,13]. Autonomic dysreflexia is clinically manifested as an increase in baseline SBP of >20 mmHg, and may be associated with pupillary dilation (blurred vision), upper body sweating, cutis anserina (goosebumps), pounding headache and flushing above the level of SCI, often with reflex bradycardia, although the initial presentation may demonstrate tachycardia [4,12,14]. The most common cause for life-threatening AD is bladder dysfunction, including bladder distension, bladder or kidney stones, blocked indwelling bladder catheter, catheterization itself, detrusor sphincter dyssynergia, shock wave lithotripsy, urinary tract infection UTI), or urological instrumentation [12].

## 4. Assessment

**Figure 2:**
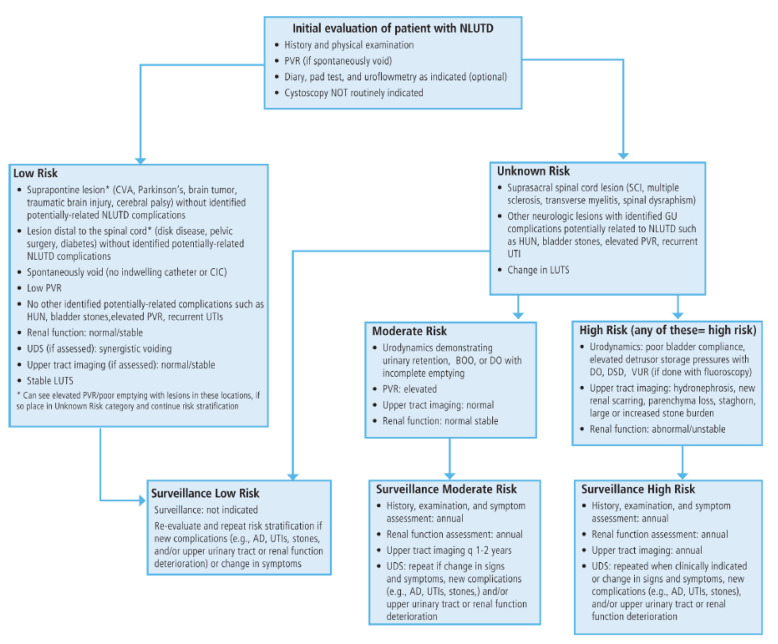
AUA/SUFU neurogenic lower urinary tract dysfunction risk stratification flow chart. Adapted with permission from Ref. [15]. 2021, Ginsberg et al.

The presentation and management of neurogenic bladder vary based on the level of SCI; however, it is important to note that even patients with similar levels of SCI may not present in the same way. For these reasons, the American Urological Association (AUA) and Society of Urodynamics, Female Pelvic Medicine and Urogenital Reconstruction (SUFU) Guidelines on Adult Neurogenic Lower Urinary Tract Dysfunction (NLUTD) for Diagnosis and Evaluation [15], as well as Treatment and Follow-up [16], have recently been provided. History and physical examination, urinalysis, post-void residual (PVR), bladder diaries, non-invasive tests, and invasive tests are all important in the initial evaluation of NLUTD for risk and stratification (Figure 2).

## 5. Initial Evaluation History and Physical Examination and Urinalysis

Medical comorbidities including neurogenic restrictive and obstructive lung disease, neurogenic bradycardia, neurogenic orthostatic hypotension, adaptive myocardial atrophy, AD, venous thromboembolism, neuropathic pain, spasticity, neurogenic bowel, erectile dysfunction, osteoporosis, heterotopic ossification, and metabolic syndrome should be considered, as they may influence bladder assessment and management strategies. Social environment and equipment should be considered. Previous and current bladder management strategies should be clarified. Dexterity determination will be important for the individual to independently perform clean intermittent catheterization (CIC); lack of appropriate hand function will likely require an attendant to assist, or less favorable management with an indwelling catheter. Those with a history of ongoing or intermittent AD and problematic spasticity related to bladder management should be considered high risk until proven otherwise [12]. Bladder morbidity events including recent AD, UTI, hematuria, incontinence, bladder or renal calculi should be discussed, including emergency department visits and or hospitalizations. Medication allergies and use of opioids, anticholinergics, antipsychotics, antidepressants, and alpha-adrenergic agents should be documented as they may influence bladder management. As part of the physical examination, ISNCSCI [11] and ISAFSCI [4] should be verified for level of injury (LOI) and completeness of SCI, including the American Spinal Injury Association Impairment Scale (AIS: A, complete without sacral sparing; B, incomplete with sacral sparing and sensory sparing below the level of injury (BLOI); C, incomplete with sacral sparing and antigravity motor spared in the majority of muscle groups BLOI; D, incomplete with sacral sparing and antigravity strength in the majority of muscle groups BLOI; E, incomplete with sacral sparing and normal motor and sensory findings BLOI) [11]. It should be noted that persons with AIS E SCI may still have hyperreflexia, AD and spasticity despite normal motor and sensory function. Hip adductor tone and hyperreflexia may indicate difficulty with perineal hygiene. Rectal tone, voluntary contraction of the anal sphincter, and the presence of bulbocavernosus reflex will assist in distinguishing UMN from LMN bladder.

## 6. Non-Invasive Testing

For those who indicate they are voluntarily voiding, a PVR should be obtained, as elevated PVR may indicate detrusor underactivity, bladder outlet obstruction (BOO), or both [16]. In select patients, a 3-day bladder diary can be used to help provide exact urinary symptoms, including bladder volumes, frequency, and urinary incontinence as well as episodes of AD [16]. Similarly, the pad test is a noninvasive, inexpensive tool to determine and quantify urinary incontinence by weighing a urinary pad or diaper before and after a 24-h period. Uroflowmetry measures the volume of urine voided per second and is a simple test that integrates bladder function and bladder outlet function over time during a voiding event [16]. However, a normal uroflowmetry study does not exclude a co-existing abnormality. Urinalysis should be obtained, as well as some assessment of renal function that can include a renal panel, 24-h creatinine clearance or cystatin C, which may be superior as it is independent of muscle volume, which is likely to be depleted after SCI.

## 7. Urinary Tract Infections

Patients with NLUTD who experience urinary tract infections (UTIs) are at higher risk of complications, such as renal complications and increased mortality [15,17]. Unfortunately, symptoms of a UTI in a patient with SCI may not present with classic UTI symptoms as observed in the general population. While classic symptoms may present, such as fever, malaise, and changes in the voiding pattern including increased incontinence or frequency, other presenting signs may be episodes of AD, resulting in increased sweating and lower extremity muscle spasms [17,18] Therefore, a high index of suspicion is needed in this patient population. In our experience, patients who have longstanding neurologic disorders with recurrent UTIs may identify unusual symptoms that reliably indicate a UTI. These symptoms should be taken seriously by clinicians and an appropriate workup should be performed. Early intervention on a UTI in this patient population can prevent life-threatening infections in this at-risk population.

However, this must be balanced with the overtreatment of patients with asymptomatic bacteriuria to prevent multidrug antibiotic resistance [15,19]. When patients are asymptomatic, as in the general population, bacteriuria should not be treated. In the setting of a symptomatic UTI, an extended course of antibiotics is recommended with antimicrobial susceptibility testing. In patients with febrile UTIs that are nonresponsive to appropriate antibiotic therapy, or have not had routine renal imaging, it is appropriate to include an upper tract imaging study as part of the workup to evaluate for nephrolithiasis or anatomic abnormalities [15,17].

The management of recurrent UTIs without abnormalities of the upper tract can be challenging in this at-risk population. Cranberry pills have been shown to be ineffective in preventing UTIs in patients with NLUTD; therefore, they should not be recommended [15]. There are studies that indicate patients performing CIC have may experience less frequent UTIs than those with an indwelling catheter and having patients with recurrent UTIs switch to CIC when feasible is a reasonable intervention [16]. Daily oral antibiotic prophylaxis is a commonly used intervention for the treatment of recurrent UTIs, although studies suggest that this is more effective in reducing asymptomatic bacteriuria than symptomatic UTIs [19]. Continuous nitrofurantoin use for more than ninety days in patients with a history of SCI was shown to be associated with fewer UTIs in one year, along with no significant change in multi-drug resistant organisms when compared to the control [20]. Current guidelines from the AUA/SUFU recommend against daily oral prophylaxis in patients with indwelling catheters, due to the high risk of development of antibiotic resistance without a significant decrease in symptomatic UTIs. However, daily oral prophylaxis may be considered in patients who perform intermittent catheterization, as there is weak evidence to support a reduction in symptomatic UTIs in this population, although careful discussion with the patient of the risk of antibiotic resistance is needed. Finally, antibiotic bladder instillations in patients who perform intermittent catheterization may help reduce recurrent UTIs, although this benefit is not observed in patients with indwelling catheters.

## 8. Upper Tract Imaging

Renal and bladder ultrasounds can be used to determine a PVR volume, and more critically to evaluate upper tract dysfunction. An ultrasound that demonstrates hydroureter or hydronephrosis without underlying upper tract disorders indicates that the storage pressures of the bladder are unacceptably high. This can be a result of either prolonged incomplete bladder emptying, where large, undrained bladder volumes cause excess pressure, or there can be an underlying pressure disorder of the bladder. Namely, this involves either high compliance (high△ volume/△ pressure) of the bladder, or low compliance (low△ volume/△ pressure), as observed in DSD. Untreated, this increased pressure can lead to upper urinary tract damage including hydronephrosis, pyelonephritis, and renal failure [6,21]. Therefore, early recognition of these processes is essential, and renal ultrasound is an important screening tool [15,17,22]. Of note, while those persons at moderate-risk for NLUTD only need to have renal imaging once every 1–2 years, annual upper tract imaging is recommended for those at high risk [15].

## 9. Invasive Testing

### 9.1. Urodynamics

Urodynamic studies allow the assessment of lower tract function, including evaluation of detrusor compliance and voiding pressures [8,15,23]. This study involves the placement of a urethral catheter, as well as an abdominal catheter (placed either in the rectum or the vagina) that allows for the interpretation of detrusor pressures during filling and voiding. This can be combined with fluoroscopic images to provide anatomic details, such as bladder diverticuli, vesicoureteral reflux, bladder outlet obstruction, and stones [8,15,23]. Urodynamic testing is critically important to diagnose conditions that predispose the patient to upper tract damage, such as poor bladder compliance, DSD, and bladder outlet obstruction. Therefore, it should optimally be completed within three months of SCI [24]. In suprasacral SCI, where often the underlying pathology is a loss of inhibition of the micturition reflex, this will appear on urodynamics as uninhibited detrusor contractions, whereas in a sacral/infrasacral injury, the detrusor muscle will most likely be either underactive or atonic [25]. Of note, clinicians must hemodynamically monitor individuals at risk for AD throughout the urodynamic study, and the study should be terminated and bladder drained if AD occurs. If hemodynamic improvement does not occur with this maneuver, pharmacotherapy should be considered [15]. The bladder volume and pressure at which AD occurred should be documented and considered when providing treatment recommendations. For patients with chronic SCI who are at moderate or high risk NLUTD and experience new signs, symptoms or complications, e.g., recurrent UTIs, stones, AD, multichannel urodynamics may be performed to determine the etiology and new treatment strategy [15].

### 9.2. Cystoscopy

Urethrocystoscopy should be used to evaluate the lower urinary tract in persons with NLUTD who have concomitant hematuria, recurrent UTIs, suspected anatomic anomaly or stones, but not routinely performed as screening or surveillance [15]. Although patients with a history of SCI and chronic indwelling catheter use have been shown to have a higher risk of developing bladder cancer in some studies [26,27], recent systematic reviews have demonstrated that cystoscopy and cytology are poor screening tests for bladder cancer in NLUTD, and those without signs and symptoms listed above should not routinely be screened [15,28,29].

## 10. Management Goals

### 10.1. Urinary Catheterization

A critical goal in the management of neurogenic bladder in SCI is preventing renal deterioration. Recurrent complicated urinary tract infections can progress to significant renal disease, and the prevention of these infections is an important facet to management. Prior to the use of clean intermittent catheterization (CIC), also known as self-catheterization, renal disease was one of the major forms of mortality for these patients [30]. CIC is regarded as the best practice for preventing UTIs compared to other methods, since it does not involve indwelling long-term foreign body use and allows for more natural bladder filling and voiding cycles [16,30]. By comparison, both indwelling urethral catheter (IUC) and suprapubic catheters (SPC) remain in the body for several weeks before being changed. An IUC is positioned through the urethra into the bladder similarly to a CIC but is attached to a bag outside of the body for long-term urine collection. The urine continuously drains into this bag, which is emptied when full. A SPC is inserted into the bladder through a small hole in the abdomen above the pubic bone and is similarly attached to a bag into which urine is drained. The advantages to the SPC are that it does not risk urethral trauma, strictures or catheter-induced urethritis, does not interfere with sexual function, and is less likely to be soiled in the event of bowel incontinence [31]. Following SCI, an IUC is used for initial management. Once the patient is medically stable, they can be taught and transitioned to CIC [24]. While the early use of CIC may decrease the risk of infection, studies have shown that it does not significantly affect complication rates at 1-year post-SCI [32]. Even though CIC remains the gold standard for persons with SCI and is well accepted [16,33], another study showed that up to 70% of patients transition to another method over time, due to inconvenience and incontinence [28]. Surprisingly, CIC and SPC were shown to have a comparable risk of UTI in a scoping review that also indicated similar themes with regard to quality of life, including the desire to discuss sexual limitations, altered body image, and acknowledged resistance to catheter insertion [34].

### 10.2. Crede, Valsalva

Bladder expression for persons with sacral and infrasacral lesions may be safely achieved by manually increasing bladder pressure so that it can overcome resistance to generate a flow with collection by an external (condom) catheter [35]. Those individuals with LMN/low pressure bladders can also use a Valsalva maneuver, in which they bear down and engage their abdominal muscles to facilitate voiding [36].

### 10.3. Reflex Voiding

Bladder reflex triggering is the induction of bladder contraction through the non-physiologic activation of Aδ stretch and C pain receptors by tapping the abdomen seven to eight times every three seconds until a flow is generated [35]. A risk of this method is the concurrent but unintended contraction of the external urethral sphincter, causing detrusor sphincter dyssynergia; a complication that occurred in 46% of the urodynamic studies on persons with SCI using this method of bladder management [37]. This is considered a dangerous practice that can result in AD, vesicoureteral reflux, renal deterioration, UTI, lithiasis, and loss of bladder compliance, and should be discouraged by SCI clinicians [35,37].

## 11. Pharmacological Interventions

### 11.1. Antimuscarinics

Several oral therapies are used in the management of neurogenic bladder in SCI patients. The most widely used class of medication remains anticholinergic agents. By inhibiting acetylcholine, bladder contractions are also inhibited. This can improve symptoms of incontinence, urgency, and frequency. Some anticholinergic drugs commonly used include oxybutynin, propantheline, and tolterodine [16,38,39]. Oxybutynin is the most versatile of these options, available in immediate and extended release as well as a patch. Side effects of this class of medications include dry mouth and eyes, constipation, headache, and tachycardia [16,38,39].

### 11.2. α1 Adrenergic Antagonists

Alpha adrenergic antagonists work by relaxing the bladder neck and decreasing outflow resistance. Drugs such as tamsulosin and terazosin have been shown to decrease incontinence, increase bladder capacity, improve bladder emptying and reduce the incidence of AD [16,40,41]. Side effects include postural hypotension and dizziness, as well as erectile dysfunction in tamsulosin specifically [35].

### 11.3. Beta 3 Adrenergic Agonist

Mirabegron is a β_3_ adrenergic agonist that has been used to effectively treat overactive bladders. The activation of β_3_ adrenergic receptors causes detrusor relaxation with reduced pressure and, therefore, improved bladder storage. It has been most thoroughly studied in males with LUTD due to benign prostate hyperplasia [7,42], and shows promise in SCI populations, with reduced urinary incontinence, increased bladder capacity and reduced cognitive side effects compared to anticholinergic agents [43,44,45]. It is associated with a mild increase in heart rate and blood pressure and may be avoided in patients with severe hypertension [7,42]. Combination therapies using mirabegron with an alpha blocker have also shown to be effective in patients who have overactive bladder that is not able to be controlled with the use of an alpha blocker alone [7]. Vibegron is a newer β_3_ adrenergic agonist that has also been shown to be effective in controlling overactive bladder alone or in combination with an antimuscarinic agent, such as tolterodine [46]. Notably for SCI patients, vibregon has demonstrated efficacy in a trial of spina bifida patients experiencing neurogenic bladder [47].

### 11.4. Bladder Chemodenervation

Botulinum toxin is produced by Clostridium botulinum and blocks synaptic activity at the neuromuscular junction through decreasing the release of acetylcholine [48,49,50]. The toxin blocks the synaptosomal associated receptor (SNARE) complex binding to the axon terminal wall and preventing exocytosis of the acetylcholine vesicle, thereby blocking muscle contraction [49]. The injection of botulinum toxin (typically onabotulinumtoxinA, or Botox^®^, Allergan USA, Inc., Madison, NJ, USA) into the bladder wall is highly effective in relaxing the detrusor muscle to treat OAB and incontinence [16,50,51,52]. This is a minimally invasive approach that is reversible, and the long term injection has been shown to be effective for up to 9 months [50]. The major risks for this procedure include autonomic dysreflexia during the procedure (prophylaxis is recommended for those at risk), urinary retention, and systemic effects of botulism toxicity including weakness and respiratory depression [16,50].

## 12. Surgical Management

Surgical management of neurogenic bladder is needed when less invasive interventions fail. The patient’s LUT dysfunction symptoms, level of injury, and diagnostic testing results must be taken into consideration when planning for surgical intervention. For patients with primarily storage dysfunction symptoms, bladder augmentation may be an option. For those with voiding dysfunction symptoms, external urethral sphincter incision and transurethral resection of prostate may all be considered. In both cases, sacral neuromodulation and urinary diversion may be considered.

### 12.1. Sphincterotomy/Intraurethral Stents

For male patients who are able and willing to use a condom catheter, external urethral sphincterotomy and/or intraurethral stents may be useful to allow for adequate bladder emptying in those otherwise generating high bladder pressures due to DSD. Unfortunately, there is a high failure rate with external sphincterotomy procedures, with 78% of patients having to undergo up to three repeat sphincterotomy procedures [16,53]. Unfortunately, intraurethral stents do not seem to be equal to or superior to a sphincterotomy. In suprasacral SCI patients who underwent intraurethral stents over sphincterotomy, urinary tract stones and intraurethral stent migration were the most common complications, with 15 out of 33 stents removed by 18 months on average [54].

### 12.2. Sacral Anterior Root Stimulation (SARS) and Sacral Neuromodulation

The AUA/SUFU Guidelines indicate clinicians should not offer sacral neuromodulation to NLUTD patients with SCI due to the high variability in bladder dysfunction in this population [16]. In sacral injuries where the detrusor is areflexic, sacral anterior root stimulation (SARS) might be used to help stimulate the emptying of the bladder. A stimulator is placed intradurally at the sacral anterior root to stimulate the sacral pelvic nerve to cause detrusor contraction, and a posterior sacral root rhizotomy is also performed to prevent detrusor hyperreflexia by removing the sensory afferent pelvic nerves [55]. Sacral neuromodulation might be an option in patients who have failed all other management options, as it is a less-invasive approach than SARS, where the electrode is placed extradurally, and there is no need for posterior root rhizotomy [55]. Incomplete sacral spinal cord injuries result in a hypotonic detrusor muscle and subsequent incomplete bladder emptying. In a study carried out on eight participants with non-obstructive urinary retention due to SCI, catheterization use was decreased and urodynamic studies showed an increase in mean maximum flow rate, an increase in mean bladder capacity, and a decrease in maximum detrusor pressures [56].

### 12.3. Bladder Augmentation

Bladder augmentation is used for patients with high bladder pressures and poor detrusor compliance that is refractory to oral therapies and bladder chemodenervation [57]. A portion of the bowel, anywhere from the ileum to colon, is used to enlarge the bladder to create a larger reservoir. This is often combined with the creation of a catheterizable stoma, either as an appendiceal vesicostomy (Mitrofanoff) [58]. or ileal vesicostomy (Monti) [59]. Patients who underwent surgery, including bladder augmentation and urinary diversion, were found to experience fewer urinary symptoms, fewer bladder management difficulties, and improved satisfaction with their urinary function [60]. Some potential complications include leakage of anastomosis, infection, UTI, bowel obstruction, and ileus [57].

### 12.4. Continent Urinary Diversion

Two types of urinary diversion surgical techniques exist, the continent and incontinent diversion. The continent urinary diversion procedure allows for urine to collect in the body and requires the ability to intermittently remove the urine via catheterization or urination and is preferred to incontinent techniques [57]. The two types of continent urinary diversion include a continent cutaneous reservoir and a bladder substitute. A continent cutaneous reservoir attaches a portion of the bowel to the ureters and allows for urine to collect in the intra-abdominal reservoir that is connected to a stoma [57]. The patient may catheterize the stoma opening to remove the urine collected. Another type of continent cutaneous reservoir is the Mitrofanoff appendicovesicostomy where, instead of the bowel functioning as a reservoir, the appendix is used instead [58]. As an alternative to the appendix, a procedure known as the Yang-Monti ileovesicostomy uses a portion of the small bladder instead when the appendix is either non-functional or absent [61]. In a bladder substitute procedure, the portion of bowel is connected to the ureters and urethra, thereby replacing the bladder [62].

### 12.5. Incontinent Urinary Diversion

The incontinent urinary diversion creates a channel to constantly redirect the urine to an intra-abdominal pouch connected to a stoma, or urostomy. Indications for an incontinent urinary diversion procedure include impaired renal function and an inability to perform catheterization [18]. There are several types of incontinent urinary diversion, including the ileal conduit, cutaneous ureterostomy, ileovesicostomy, and ureteroileostomy. The ileal conduit takes a part of the ileum and functions as a passageway for the urine from the ureters to the opening on the skin, or stoma. The ureterostomy allows for the passage of urine from the ureters directly to the stoma, but is rarely used because stricture and stenosis rates are high [57]. The ileovesicostomy takes an isolated part of the ileum and connects one end to the bladder, instead of the ureters, and the other end to the stoma. This can be chosen over an ileal conduit procedure due to risk of uretero-intestinal strictures [63].

## 13. Surveillance

The management of patients with neurogenic bladder following spinal cord injury necessitates a multidisciplinary care approach, due to the complicated nature of the dysfunction. Patients should be followed closely by their SCI specialist physician and urology for management. Recent guidelines for assessment [15] and treatment [16] of NLUTD have identified those with SCIs of unknown risk until the initial evaluation has been completed, with recommendations for annual H and P, renal function assessment, upper tract imaging and repeat urodynamic studies when clinically indicated for those at high risk, i.e., those with poor bladder compliance, demonstrated hydronephrosis or high stone burden, and those with abnormal renal function [15]. Those at moderate risk (urinary retention, bladder outlet obstruction and/or detrusor overactivity), are recommended to have annual H and P and renal function assessment, with upper tract imaging every 1–2 years; urodynamics should only be repeated if there are changes in signs, symptoms or new complications. Those at low risk spontaneous voiding with low PVR, normal/stable renal function, normal stable upper tract imaging and synergistic voiding will only need assessment if there is a change in signs, symptoms or new complications. One should consider the patient’s quality of life with their symptoms, as well as their risk of further complications. Finally, cystoscopy should be reserved for those individuals with NLUTD who have concomitant hematuria, recurrent UTIs, or suspected anatomic anomaly (e.g., strictures and false passages) [15].

## Figures and Tables

**Table 1:** Innervation of the urinary tract.

Anatomic Location	Sympathetic NS (Norepinephrine)	Parasympathetic NS (Acetylcholine)	Somatic Innervation (Acetylcholine)
Kidney	Renal plexus	Renal plexus	NA
Ureters	T12–L2	S2–S4	Superior hypogastric plexus
Bladder Dome	Hypogastric Nerve (T10–L2) on β3 Adrenergic Receptors	Pelvic Nerves (S2–S4) on M_3_ ACH Receptors	NA
Bladder Neck	Hypogastric Nerve (T10–L2) on α_1_ Adrenergic Receptors	Pelvic Nerves (S2–S4) on M_3_ ACH Receptors	NA
External Urethral Sphincter/Pelvic Floor Muscles	NA	NA	Pudendal Nerve (S2–S4) on Nicotinic ACH Receptors

**Table 2:** Spinal cord level of injury and associated dysfunction.

Spinal Cord Lesion	Bladder and External Urethral Sphincter Dysfunction
Suprasacral Upper Motor Neuron (UMN) Injury	Storage Dysfunction Detrusor Overactivity with decreased Detrusor Compliance Hyperreflexic External Urethral Sphincter Detrusor Sphincter Dyssynergia (DSD) Upper Urinary Tracts at Risk due to High Detrusor Pressure (>40 cm H_2_O) Urinary incontinence
Mixed: UMN and/or LMN Injury	Storage Dysfunction Detrusor Overactivity or Areflexia (increased or decreased Detrusor Compliance) Hyperreflexic or Flaccid External Urethral Sphincter Detrusor Sphincter Dyssynergia (DSD) or Detrusor Sphincter Areflexia Upper Urinary Tracts May be at Risk due to High Detrusor Pressure (>40 cm H_2_O) Urinary incontinence
Sacral/Infrasacral Lower Motor Neuron (LMN) Injury	Voiding Dysfunction Areflexic Detrusor/Flaccidity with increased Detrusor Compliance External Urethral Sphincter Areflexia/Flaccidity Overflow Urinary Incontinence

## Data Availability

Not applicable.

## Abbreviations

Autonomic nervous system (ANS), autonomic dysreflexia (AD), International Standards to determine remaining Autonomic Function after Spinal Cord injury (ISAFSCI), International standards for Neurological Classification of Spinal Cord Injury (INCSCI), neurogenic lower urinary tract dysfunction (NLUTD), neurogenic orthostatic hypotension (NOH), parasympathetic nervous system (PNS), spinal cord injury (SCI), sympathetic nervous system (SNS) and sympathetic skin response (SSR).
